# Clinical predictors of treatment effectiveness in late onset schizophrenia and schizophrenia-like psychosis

**DOI:** 10.1192/j.eurpsy.2024.800

**Published:** 2024-08-27

**Authors:** V. Pochueva, T. Safarova, V. Sheshenin

**Affiliations:** ^1^FSBSI “Mental Health Research Center”, Moscow, Russian Federation

## Abstract

**Introduction:**

Clinical features and structural changes in the brain of patients with late-onset schizophrenia and schizophrenia-like psychosis are important in predicting the effectiveness of treatment.

**Objectives:**

Identification the dependence of effectiveness of psychopharmacotherapy on the clinical features and structural brain changes in late-onset schizophrenia and schizophrenia-like psychosis.

**Methods:**

111 patients, age from 52 to 89 years with ICD-10 diagnosis F20, F25, F22.8, F06.2 were investigated for 28 days. Clinical, psychometric methods with PANSS, CGI, HAMD, CDSS, MMSE scales were used. MRI/CT were performed. Effectiveness of treatment was measured in two ways: 1. Percentage ratio of reduction in total scores to the 1^st^ value of scales. 2. The number of responders (patients with a decrease in PANSS by 30% or more).

**Results:**

The effectiveness of treatment in the overall group was 29,4% on the PANSS scale (from -13,6% to 77,2%). The greatest effectiveness was on subscale of positive syndromes (34,9%), the lowest – on the subscale of negative syndromes (18,6%). The number of responders (R) was 43 patients (38,7%), non-responders(NR) – 68 patients (61,3%). The responder group was characterized by a greater severity of acute psychosis before the begging of treatment. Early insomnia, excitement and anxiety, decreased appetite, valuated by HAMD scale were significantly more pronounced. Treatment effectiveness had negative correlates (p<0,05) with number of acute attacks, number of hospitalizations and the duration of current attack. The predominance of negative symptoms has a negative correlation with effectiveness by PANSS and CGI scales. According to the results of MRI/CT examination, cortical atrophy, vascular changes and leucoaraiosis were more often represented in NR group.

**Conclusions:**

The connection between the effectiveness of treatment and the clinical and psychopathological features and structural changes in late onset schizophrenia and schizophrenia-like psychosis was shown.

**Disclosure of Interest:**

None Declared

